# Bat-associated Rabies Virus in Skunks

**DOI:** 10.3201/eid1208.051526

**Published:** 2006-08

**Authors:** Mira J. Leslie, Sharon Messenger, Rodney E. Rohde, Jean Smith, Ronald Cheshier, Cathleen Hanlon, Charles E. Rupprecht

**Affiliations:** * Washington State Department of Health, Shoreline, Washington, USA;; † California Department of Health Services, Richmond, California, USA;; ‡ Texas State University-San Marcos, San Marcos, Texas, USA;; § Centers for Disease Control and Prevention, Atlanta, Georgia, USA;; ¶ Arizona Department of Health Services, Phoenix, Arizona, USA

**Keywords:** Rabies, surveillance, bat, skunk, molecular epidemiology, wildlife reservoirs, RT-PCR, phylogenetic analysis, cross-species infection, dispatch

## Abstract

Rabies was undetected in terrestrial wildlife of northern Arizona until 2001, when rabies was diagnosed in 19 rabid skunks in Flagstaff. Laboratory analyses showed causative rabies viruses associated with bats, which indicated cross-species transmission of unprecedented magnitude. Public health infrastructure must be maintained to address emerging zoonotic diseases.

In North America, >90% of cases of rabies in animals occur in wildlife (*1*); several mammalian taxa harbor characteristic rabies virus variants (RABVV). In Arizona, skunks (*Mephitis mephitis*) and gray foxes (*Urocyon cinereoargenteus*) maintain independent rabies enzootic cycles, and in indigenous bats, rabies has been diagnosed in 14 of 28 species (Arizona Department of Health Services, unpub. data). Although skunks live throughout Arizona, until 2001, rabid skunks had been found only in the southeastern quadrant of the state.

In the United States, bat RABVV are a source of infection for humans and other mammals (*2**–**8*). Typically, interspecies infection produces a single fatal spillover event; secondary transmission has rarely been observed. Antigenic typing of rabid carnivores in Arizona from 1996 through 2000 identified bat RABVV in 1 domestic dog and 2 gray foxes. This report describes the largest documented rabies epizootic among terrestrial mammals infected with bat RABVV, with perpetuated animal-to-animal transmission. Coincident with the zoonotic disease significance, this report provides contemporary insight into pathogen evolution (*9*).

## The Study

**Figure 1:**
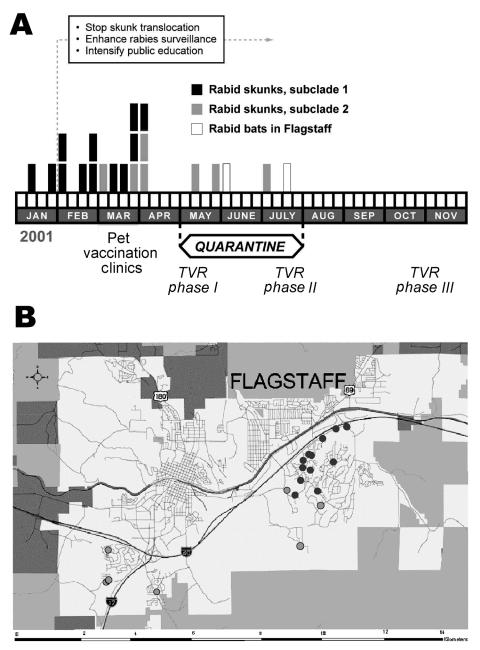
Temporal and geographic distribution of rabies outbreak in Flagstaff, Arizona. A) Timeline and control measures. TVR: trap, vaccinate, release program. B) Geographic location of rabid skunks (dark gray dots = subclade 1, light gray dots = subclade 2).

In January 2001, a homeowner contacted Flagstaff Animal Control about a dead skunk. Although no human had been exposed to the skunk, tissues were submitted to the Arizona State Health Laboratory, where rabies was diagnosed. This skunk was the first rabid terrestrial wild carnivore reported from the area. The Texas Department of State Health Services subsequently identified an RABVV associated with bats in tissues sent for antigenic characterization. From January through April, 14 more skunks, dead or exhibiting abnormal behavior, were found throughout a large residential subdivision within 4 km of the initial case. All were infected with the same bat RABVV. From April through July, 4 more skunks infected with bat RABVV were identified ≈9 km west of the initial focus (Figure 1). Control measures included prohibiting relocation of nuisance skunks, comprehensive public education, pet rabies vaccine clinics, and a 90-day emergency quarantine requiring pets to be leashed or confined and vaccinated (Figure 1). Additionally, 217 urban skunks were vaccinated and marked with ear tags during a 6-month phased program of trap, vaccinate, and release.

In Flagstaff and the surrounding county, during the decade before this epizootic, 2 rabid bats, on average, were reported each year. During the epizootic, 218 animals were submitted for rabies testing (Table). Rabies was confirmed in 19 (13%) of 145 tested skunks and 2 (9%) of 22 tested bats. Although most (18 [95%]) of the rabid skunks were identified and reported by lay citizens, no contact between these skunks and humans or domestic animals was reported.

**Table:** Animals from Flagstaff submitted for rabies diagnosis, January–July, 2001

Animal	Scientific name	No. submitted	No. rabid
Skunk	*Mephitis mephitis*	145	19
Bat	(Multiple spp.)	22	2
Domestic cat	*Felis domesticus*	12	0
Gray fox	*Urocyon cinereoargenteus*	9	0
Domestic dog	*Canis familiaris*	9	0
Squirrel	Species unknown	8	0
Coyote	*Canis latrans*	4	0
Raccoon	*Procyon lotor*	2	0
Porcupine	*Erethizon dorsatum*	2	0
Prairie dog	*Cynomys ludovicianus*	2	0
Badger	*Taxidea taxus*	1	0
Opossum	*Didelphis virginiana*	1	0
Bobcat	*Lynx rufus*	1	0
Total		218	21

Local baseline population estimates were not available to indicate whether skunk demography affected disease attributes. Synchronous with this outbreak, independent epizootic activity caused by well-established skunk RABVV was documented in southern Arizona, which suggests that regional skunk epizootiologic dynamics were similarly affected. Skunks' seasonal behavior may have contributed to transmission events. This epizootic was initially recognized when a dead skunk appeared in a snow-covered backyard, during a season when skunks are in communal dens. Given an incubation period of 2 months, most transmission would have occurred between late autumn (when skunks are in their dens) and late winter (when they are mating).The Flagstaff epizootic peak coincided with nationwide seasonal trends of rabid skunks (*1*). Enhanced postepizootic surveillance in Flagstaff did not detect additional rabid terrestrial mammals for the next 3 years. However, in 2004, a total of 5 skunks found in the initially affected east Flagstaff neighborhood and 1 fox 28 km south of Flagstaff were infected with the same bat RABVV (*10*).

**Figure 2:**
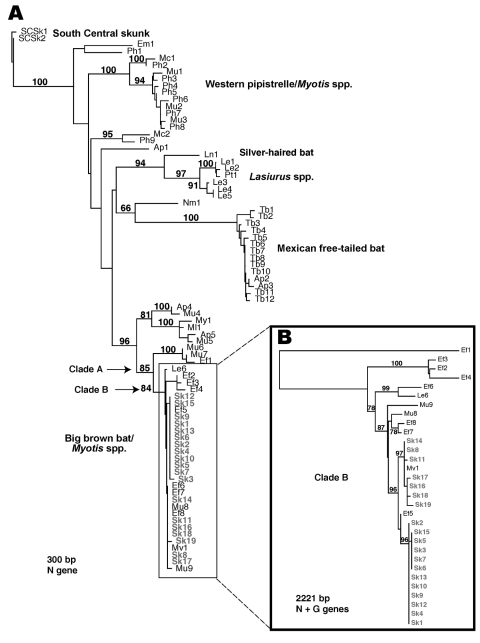
A) Phylogenetic tree of the 19 rabid skunk isolates and representative samples of known rabies virus variants (RABVV) from Arizona based on 300 bp of the nucleoprotein (N) gene (GenBank accession no. AY170226–304). B) Detailed analyses of clade including all 19 skunk isolates (clade B) based on 2221 bp of the N and glycoprotein (G) genes (GenBank accession no. AY170397–438). Phylogenetic analyses used PAUP* software (version 4.0b2, Sinauer Associates, Sunderland, MA, USA; 2000] using the neighbor-joining search algorithm (minimum evolution) with maximum likelihood to estimate Ti:Tv ratio and nucleotide base frequencies (HKY85 model). Numbers at tree nodes indicate nonparametric bootstrap proportions based on 1,000 replicates.

Viruses isolated from the rabid skunks exhibited monoclonal antibody patterns similar to RABVV associated with big brown (*Eptesicus fuscus*) and *Myotis* bats in the western United States (*11*). These are among the most abundant bat species in Arizona and often roost in houses and outbuildings; however, no bat colonies were found in association with any of the rabid skunks. Restriction digests of PCR amplicons from the rabid skunks did not match patterns known for RABVV from North American terrestrial reservoirs (*12*). Phylogenetic analysis of a 300-bp region of the N gene showed that the Flagstaff skunk RABVV was identical (100%) to Arizona bat RABVV (Table A1, Figure 2A), and differed by 22% from skunk and gray fox RABVV. A monophyletic clade (clade A) of 8/8 big brown, 5/14 *Myotis*, and 1/6 southern yellow (*Lasiurus ega*) bats shared >95% identity with Flagstaff skunk RABVV. An additional 44 samples, representing 11 bat species, differed by >8% from Flagstaff skunk RABVV.

An analysis of clade A, which incorporates N and G genes, indicated that the Flagstaff skunk RABVV were more closely related to 2 bat RABVV (*E. fuscus* from Coconino County, *M. velifer* from Maricopa County) collected in 1999 and 1997 than to the 2 bat RABVV collected locally during the outbreak. In clade B, subclade 1 RABVV were collected from January through early April from the northeastern region of the outbreak, whereas subclade 2 RABVV were collected from early March through July from the southeastern and western regions of the outbreak (Figure 1). However, phylogenetic data do not support a wavelike spread from northeast to west because this would require nesting of subclade 1 within subclade 2. In contrast, both subclades exhibit independently derived mutations. East-to-west epizootic movement of RABVV within subclade 2 (sk16–19 form a monophyletic clade nested within subclade 2) during April is supported by the data and may be related to dispersal of infected skunks along river corridors or translocation by humans. One person reported trapping, moving, and releasing a skunk before the outbreak was known in the community. Alternatively, apparent shifts may be an artifact of intensified public awareness and reporting. Lack of sampling in the uninhabited forest between the eastern and western foci limits our ability to discriminate among these hypotheses.

## Conclusions

This is the largest recorded cluster of bat RABVV infection in terrestrial mammals. Investigation of this novel outbreak showed evolution in action with the emergence of an RABVV that successfully adapted from Chiroptera to Carnivora. Previously documented clusters involving 3–4 to terrestrial mammals infected with a single insectivorous bat rabies virus variant did not corroborate sustained transmission (*12*). Although >1 skunk may have been exposed to a single rabid bat, it is highly unlikely that each skunk was exposed to the same bat or that multiple bat-skunk exposures occurred. We could not ascertain the complete scope of this outbreak or whether it was the index event. Phylogenetic analyses support the evolution of 2 independent lineages, suggesting establishment for months or years. Additionally, virus isolation from salivary glands of 5 affected skunks and the reappearance of rabid skunks with the same RABVV in 2004 support the probability of independent transmission.

The recognition of this epizootic can be credited to a coordinated laboratory-based disease surveillance program to monitor sick and dead wildlife for potential zoonoses (plague, tularemia, rabies) even in situations lacking human or pet exposures. Comprehensive animal disease surveillance provides direct benefits to public health and animal health by promoting early recognition of risk and opportunities for disease control and prevention interventions.

Unpredictable health threats related to emerging zoonoses, especially those involving wildlife reservoirs, pose notable surveillance and control challenges (*13**–**15*). Recent bioterrorism initiatives emphasize integration of human and animal disease surveillance, and enhanced laboratory capacity, as essential functions in zoonosis detection (*13*). Rabies surveillance and control programs serve as historic prototypes for effective, long-term, public health programs. Quintessential zoonotic disease programs require innovative and expanded capacities, commitments to public health and veterinary laboratory infrastructure, and appropriate interagency and interdisciplinary coordination and communication.

## Appendix

**Table A1:** Representative rabies virus variants found in Arizona and included in phylogenetic analyses

CDC ID	State ID	Taxon name	Common name	Scientific name	Collection date	Collection Site
*3629*	*504318*	*Ap1*	Pallid bat	*Antrozous pallidus*	Jul 1997	Navajo
*3626*	*498674*	*Ap2*	Pallid bat	*A. pallidus*	Jul 1997	Maricopa
*3628*	*500509*	*Ap3*	Pallid bat	*A. pallidus*	Jul 1997	Wickenburg
*3925*	*390721*	*Ap4*	Pallid bat	*A. pallidus*	Sep 1995	Coconino
*3927*	*735430*	*Ap5*	Pallid bat	*A. pallidus*	Mar 1997	Tucson
*4891*	*10793*	*Ef1*	Big brown bat	*Eptesicus fuscus*	Sep 1999	Yuma
*4862*	*947906*	*Ef2*	Big brown bat	*E. fuscus*	Jun 1999	Davis AFB
*4867*	*9630*	*Ef3*	Big brown bat	*E. fuscus*	Aug 1999	Tucson
*4850*	*15171*	*Ef4*	Big brown bat	*E. fuscus*	Oct 1999	Tucson
*4871*	*99026842*	*Ef5*	Big brown bat	*E..fuscus*	Jun 1999	Coconino
*4886*	*99046548*	*Ef6*	Big brown bat	*E. fuscus*	Oct 1999	Yavapi
*5450*	*1034778*	*Ef7*	Big brown bat	*E. fuscus*	Jul 2001	Coconino
*5442*	*1026934*	*Ef8*	Big brown bat	*E. fuscus*	Jul 2001	Coconino
*4074*	*98007821*	*Em1*	Spotted bat	*Euderma maculatum*	Sep 1998	Maricopa
*3057*	*396597*	*Ln1*	Silver-haired bat	*Lasionycteris noctivagans*	Oct 1995	Maricopa
*3285*	*432420*	*Le6*	Southern yellow bat	*Lasiurus ega*	May 1996	Coconino
*3046*	*264111*	*Le1*	Southern yellow bat	*L. ega*	Aug 1993	Yuma
*3870*	*814565*	*Le2*	Southern yellow bat	*L. ega*	Oct 1997	Pima
*3284*	*447760*	*Le3*	Southern yellow bat	*L. ega*	Aug 1996	Yuma
*3050*	*374106*	*Le4*	Southern yellow bat	*L. ega*	Jun 1995	Yuma
*3347*	*395408*	*Le5*	Southern yellow bat	*L. ega*	Sep 1995	Yuma
*5422*	*141014*	*Scsk1*	Striped skunk	*Mephitis mephitis*	May 2001	Cochise
*5423*	*142140*	*Scsk2*	Striped skunk	*M. mephitis*	May 2001	Cochise
*4995*	*1001810*	*Sk1*	Striped skunk	*M. mephitis*	Jan 2001	Coconino/Flagstaff
*4998*	*1004508*	*Sk2*	Striped skunk	*M. mephitis*	Jan 2001	Coconino/Flagstaff
*5079*	*1006928*	*Sk3*	Striped skunk	*M. mephitis*	Jan 2001	Coconino/Flagstaff
*5470*	*1006403*	*Sk4*	Striped slunk	*M. mephitis*	Feb 2001	Coconino/Flagstaff
*5074*	*1009087*	*Sk5*	Striped skunk	*M. mephitis*	Feb 2001	Coconino/Flagstaff
*5075*	*1010227*	*Sk6*	Striped skunk	*M. mephitis*	Feb 2001	Coconino/Flagstaff
*5076*	*1010229*	*Sk7*	Striped skunk	*M. mephitis*	Feb 2001	Coconino/Flagstaff
*5077*	*1011591*	*Sk8*	Striped skunk	*M. mephitis*	Mar 2001	Coconino/Flagstaff
*5081*	*1012600*	*Sk9*	Striped skunk	*M. mephitis*	Mar 2001	Coconino/Flagstaff
*5080*	*1014008*	*Sk10*	Striped skunk	*M. mephitis*	Mar 2001	Coconino/Flagstaff
*5100*	*1015436*	*Sk11*	Striped skunk	*M. mephitis*	Apr 2001	Coconino/Flagstaff
*5101*	*1015687*	*Sk12*	Striped skunk	*M. mephitis*	Apr 2001	Coconino/Flagstaff
*5102*	*1015688*	*Sk13*	Striped skunk	*M. mephitis*	Apr 2001	Coconino/Flagstaff
*5103*	*1016511*	*Sk14*	Striped skunk	*M. mephitis*	Apr 2001	Coconino/Flagstaff
*5132*	*1016704*	*Sk15*	Striped skunk	*M. mephitis*	Apr 2001	Coconino/Flagstaff
*5133*	*1016707*	*Sk16*	Striped skunk	*M. mephitis*	Apr 2001	Coconino/Flagstaff
*5440*	*1023718*	*Sk17*	Striped skunk	*M. mephitis*	May 2001	Coconino/Flagstaff
*5441*	*1025813*	*Sk18*	Striped skunk	*M. mephitis*	May 2001	Coconino/Flagstaff
*5451*	*1036291*	*Sk19*	Striped skunk	*M. mephitis*	Jul 2001	Coconino/Flagstaff
*3858*	*721957*	*Mc1*	California myotis	*Myotis californicus*	Dec 1996	Pima
*3847*	*433224*	*Mc2*	Western small-footed bat	*M. ciliolabrum*	May 1996	Holbrook
*3848*	*445332*	*Ml1*	Little brown bat	*M. lucifugus*	Aug 1996	Wickenburg
*4882*	*99043079*	*Mu1*	Myotis bat	*Myotis sp.*	Sep 1999	Bullhead City
*3862*	*800694*	*Mu2*	Myotis bat	*Myotis sp.*	Jul 1997	Pima
*4887*	*99048912*	*Mu3*	Myotis bat	*Myotis sp.*	Oct 1999	Pima
*419*	*32455*	*Mu4*	Myotis bat	*Myotis sp.*	Apr 1983	Mesa
*3350*	*457788*	*Mu5*	Myotis bat	*Myotis sp.*	Oct 1996	Maricopa
*3351*	*390759*	*Mu6*	Myotis bat	*Myotis sp.*	Sep 1995	Yuma
*4881*	*99042806*	*Mu7*	Myotis bat	*Myotis sp.*	Sep 1999	Yuma
*4873*	*99038008*	*Mu8*	Myotis bat	*Myotis sp.*	Aug 1999	Eager
*4872*	*99028231*	*Mu9*	Myotis bat	*Myotis sp.*	Jun 1999	Pima
*3855*	*502675*	*Mv1*	Cave myotis	*M. velifer*	Jul 1997	Maricopa
*3852*	*489891*	*My1*	Yuma bat	*M. yumanensis*	May 1997	San Carlos
*3043*	*259807*	*Ph1*	Western pipistrelle	*Pipistrellus hesperus*	Jul 1993	Maricopa
*3860*	*747838*	*Ph2*	Western pipistrelle	*P. hesperus*	Jun 1997	Pima
*3044*	*259808*	*Ph3*	Western pipistrelle	*P. hesperus*	Jul 1993	Maricopa
*3924*	*455031*	*Ph4*	Western pipistrelle	*P. hesperus*	Sep 1996	Navajo
*2060*	*8543*	*Ph5*	Western pipistrelle	*P. hesperus*	Sep 1981	Sedona
*3863*	*805082*	*Ph6*	Western pipistrelle	*P. hesperus*	Aug 1997	Pima
*3345*	*466665*	*Ph7*	Western pipistrelle	*P. hesperus*	Dec 1996	Maricopa
*3859*	*735181*	*Ph8*	Western pipistrelle	*P. hesperus*	Mar 1997	Oro Valley
*3053*	*391577*	*Ph9*	Western pipistrelle	*P. hesperus*	Sep 1995	Coconino
*410*	*26202*	*Tb1*	Mexican free-tailed bat	*Tadarida brasiliensis*	May 1985	Riveria
*448*	*5646*	*Tb2*	Mexican free-tailed bat	*T. brasiliensis*	Aug 1982	Thatcher
*4860*	*943431*	*Tb3*	Mexican free-tailed bat	*T. brasiliensis*	May 1999	Tucson
*4857*	*821298*	*Tb4*	Mexican free-tailed bat	*T. brasiliensis*	Dec 1997	Tucson
*4889*	*99057833*	*Tb5*	Mexican free-tailed bat	*T. brasiliensis*	Dec 1999	Phoenix
*4863*	*848036*	*Tb6*	Mexican free-tailed bat	*T. brasiliensis*	Jun 1999	Tucson
*4868*	*9810071*	*Tb7*	Mexican free-tailed bat	*T. brasiliensis*	Oct 1998	Phoenix
*4864*	*949009*	*Tb8*	Mexican free-tailed bat	*T. brasiliensis*	Jun 1999	Tucson
*4866*	*949396*	*Tb9*	Mexican free-tailed bat	*T. brasiliensis*	Jun 1999	Tucson
*4865*	*949010*	*Tb10*	Mexican free-tailed bat	*T. brasiliensis*	Jun 1999	Tucson
*4847*	*10197*	*Tb11*	Mexican free-tailed bat	*T. brasiliensis*	Sep 1999	Tucson
*4884*	*99046042*	*Tb12*	Mexican free-tailed bat	*T. brasiliensis*	Sep 1999	Winslow
*3344*	*713793*	*Pt1*	Townsend's big-eared bat	*Plecotus townsendii*	Oct 1996	Pima
*3659*	*513855*	*Nm1*	Big free-tailed bat	*Nyctinomops macrotis*	Oct 1997	Yavapai
